# Evaluating the accuracy of *Listeria monocytogenes* assemblies from quasimetagenomic samples using long and short reads

**DOI:** 10.1186/s12864-021-07702-2

**Published:** 2021-05-26

**Authors:** Seth Commichaux, Kiran Javkar, Padmini Ramachandran, Niranjan Nagarajan, Denis Bertrand, Yi Chen, Elizabeth Reed, Narjol Gonzalez-Escalona, Errol Strain, Hugh Rand, Mihai Pop, Andrea Ottesen

**Affiliations:** 1 Center for Food Safety and Applied Nutrition, Food and Drug Administration, Laurel, MD USA; 2 Center for Bioinformatics and Computational Biology, University of Maryland, College Park, MD USA; 3 Biological Science Graduate Program, University of Maryland, College Park, MD USA; 4 Department of Computer Science, University of Maryland, College Park, MD USA; 5 Joint Institute for Food Safety and Applied Nutrition, University of Maryland, College Park, MD USA; 6 Center for Food Safety and Nutrition, Food and Drug Administration, College Park, MD USA; 7 Computational and Systems Biology, Genome Institute of Singapore, Singapore, 13862 Singapore; 8 Center for Veterinary Medicine, Food and Drug Administration, Laurel, MD USA

**Keywords:** Quasimetagenomics, Metagenomics, Source tracking, Listeria, Nanopore, Assembly

## Abstract

**Background:**

Whole genome sequencing of cultured pathogens is the state of the art public health response for the bioinformatic source tracking of illness outbreaks. Quasimetagenomics can substantially reduce the amount of culturing needed before a high quality genome can be recovered. Highly accurate short read data is analyzed for single nucleotide polymorphisms and multi-locus sequence types to differentiate strains but cannot span many genomic repeats, resulting in highly fragmented assemblies. Long reads can span repeats, resulting in much more contiguous assemblies, but have lower accuracy than short reads.

**Results:**

We evaluated the accuracy of *Listeria monocytogenes* assemblies from enrichments (quasimetagenomes) of naturally-contaminated ice cream using long read (Oxford Nanopore) and short read (Illumina) sequencing data. Accuracy of ten assembly approaches, over a range of sequencing depths, was evaluated by comparing sequence similarity of genes in assemblies to a complete reference genome. Long read assemblies reconstructed a circularized genome as well as a 71 kbp plasmid after 24 h of enrichment; however, high error rates prevented high fidelity gene assembly, even at 150X depth of coverage. Short read assemblies accurately reconstructed the core genes after 28 h of enrichment but produced highly fragmented genomes. Hybrid approaches demonstrated promising results but had biases based upon the initial assembly strategy. Short read assemblies scaffolded with long reads accurately assembled the core genes after just 24 h of enrichment, but were highly fragmented. Long read assemblies polished with short reads reconstructed a circularized genome and plasmid and assembled all the genes after 24 h enrichment but with less fidelity for the core genes than the short read assemblies.

**Conclusion:**

The integration of long and short read sequencing of quasimetagenomes expedited the reconstruction of a high quality pathogen genome compared to either platform alone. A new and more complete level of information about genome structure, gene order and mobile elements can be added to the public health response by incorporating long read analyses with the standard short read WGS outbreak response.

**Supplementary Information:**

The online version contains supplementary material available at 10.1186/s12864-021-07702-2.

## Background

### State of the art for pathogen typing

Rapid response, whole-genome sequencing (WGS) networks such as GenomeTrakr [1], PulseNet [2], and the National Antimicrobial Resistance Monitoring System (NARMS) [3, 4] have revolutionized the strain typing and source attribution of bacterial pathogens and antimicrobial resistance (AMR) important to human and animal health. These programs have relied primarily on high throughput short-read sequencing data generated using the Illumina MiSeq platform. Accurate strain typing of bacterial pathogens using short reads is typically accomplished with SNP (single nucleotide polymorphism) and/or MLST (multi-locus sequence typing) analyses. Both can be performed directly on the raw reads or with assemblies of the raw reads. SNP analyses quantify the number of SNPs between a set of isolates and a reference genome [5]. High resolution MLST analyses involve identifying the profile of alleles for genes in the core genome and whole genome [6, 7], cgMLST and wgMLST, respectively. Both methods can differentiate between very closely related strains of *Salmonella enterica*, *Listeria monocytogenes*, *Escherichia coli*, *Staphylococcus aureus* and many other pathogens [8–10]. However, despite providing high resolution, SNP and cgMLST/wgMLST analyses do not analyze nor require the entire genome assembly and, thus, miss aspects of genome architecture, such as the synteny of features and mobile elements with variable gene content [11].

### The assembly of genomes using short and long reads

Ideally, complete genomes would be routinely sequenced and assembled de novo from outbreak samples for strain typing analyses. However, this is not yet possible in every situation. Although short reads can be sequenced with an error rate of less than 0.1% [12], these reads are typically 250 base pairs or less in length and cannot span many genomic repeat regions, resulting in fragmented assemblies that preclude the recovery of complete bacterial genomes [13]. In contrast, long read sequencing technologies like the Oxford Nanopore platform have higher sequencing error rates (~ 13% [14, 15]), but can routinely produce reads that are over 10 Kbp, thus spanning genomic repeats and supporting the assembly of complete bacterial genomes and plasmids [16].

Although assemblies of nanopore long reads can generate genome-length contigs, they often have a large number of errors inherited from the reads. The hybrid assembly of Illumina short and nanopore long reads can remarkably improve the quality of the assemblies while maintaining syntenic contiguity [16]. A study of the assembly of several *Salmonella enterica* strains demonstrated that short read assembly followed by long read scaffolding, reconstructed genomes more accurately than using short reads or long reads alone [17]. Another study reconstructed entire genomes of Shiga-toxin producing *Escherichia coli* strains using nanopore long reads that were polished with Illumina short reads [18]; however, these assemblies had less accurate cgMLST typing compared to those using only MiSeq short reads, despite the short read polishing.

### Microbiological recovery of the target pathogen

Irrespective of sequencing technology, for applications such as the source tracking of bacterial pathogens, a fundamental challenge is the extraction of sufficient quantities of pathogen DNA to sequence in the first place. This is because pathogens frequently occur at low abundance in complex microbial communities, sometimes amongst large numbers of host cells, and/or in chemically challenging matrices. Current methods address this challenge by selective culture enrichment and pure colony isolation of the pathogens prior to sequencing and analysis. This approach however, is labor-intensive and can take days to weeks to provide sufficient DNA for sequencing. While protocols and media formulations for the enrichment of *L. monocytogenes* vary only slightly between agencies (Food and Drug Administration (FDA), International Organization of Standardization (ISO), and the United States Department of Agriculture (USDA)), in-house FDA metagenomic and quasimetagenomic analyses of timepoints along recovery continuums from different starting matrices have demonstrated that enrichment dynamics and efficiencies vary according to chemical and microbiological features of the input matrix (ie; different foods such as fresh produce, poultry, complex environmental samples, and varying initial loads (CFUs) of target pathogens) [19]. Community dynamics during all types of pathogen enrichments (e.g. *Salmonella enterica*, *Escherichia coli*, *Listeria spp.*) are still poorly understood and co-enriching non-target species often compete with pathogens of clinical significance [20].

### Metagenomics

Metagenomics is the direct sequencing of microbial communities [21] and, in theory, could replace culture enrichment for pathogen source tracking. Short read sequencing has been used extensively for metagenomics due to low error rates and high throughput, but cannot assemble many of the genomic and intergenomic repeats present in environmental DNA. In contrast, the long reads generated by nanopore sequencing platforms can resolve many of the genomic and intergenomic repeats. Recently, metagenomic studies have successfully used nanopore sequencing for rapid identification of dominant pathogens [22, 23] contributing complete assemblies for a small subset of the bacteria in the full metagenome [13, 24, 25]. However, achieving sufficient depth of coverage to assemble pathogen genomes directly from metagenomes is often prohibitively expensive.

### Quasimetagenomics

A middle ground between the direct sequencing of samples and the sequencing of isolates from selective enrichments is quasimetagenomics, the sequencing of abbreviated recovery enrichments [13, 26]. Quasimetagenomics has been used by FDA scientists since 2009 in efforts to recover pathogens from complex microbiomes such as outbreaks of *Salmonella* in tomatoes [27, 28], to better understand Latin cheese microbiota [29], to look at enrichments for *Salmonella* from cilantro [30], *E.coli* in flour [31], pathogens in seafood [32–34] and in the public health research response to the Blue Bell ice cream outbreak of 2015, which resulted in the dataset presented here [20, 26]. The first FDA ice cream work (2015) received a lot of attention in the food safety community and the quasimetagenomic approach was quickly emulated by other food safety research groups [26, 35, 36]. Many groups are moving the needle forward–demonstrating that strain level differentiation during an outbreak response can be achieved more rapidly with quasimetagenomic approaches [35, 36]. Here we build upon the first ice cream report [20] which demonstrated that a quasimetagenomic approach could recover the same quality of source tracking data much earlier than state of the art WGS approaches; and a second work which validated the bioinformatic SNP and cgMLST source tracking efficiency of the quasimetagenomic data [26]; and–presented here–the added value of GridIon long reads for circularization of genomes and plasmids.

### Integrated microbiological, molecular and bioinformatic innovations that will move the field forward

Here, we provide a detailed benchmarking analysis for assessing how rapidly and accurately a targeted pathogen, *L. monocytogenes*, can be assembled from quasimetagenomic samples using short and long read sequencing technologies. The evaluated assembly tools include those developed specifically for metagenomic assemblies (MegaHit for short read assembly, metaFlye for long read assembly, and Opera-MS for hybrid assembly) as well as popular tools developed for long read genome assembly (Canu and Redbean) and hybrid genome assembly (HybridSpades). Additionally, we evaluated the impact of polishing with three tools: Pilon, ntEdit (both were used to polish long read assemblies with short reads), and Racon (was used to polish long read assemblies with long reads). The results of this study allowed us to point out the strengths and weaknesses in currently available tools and to make recommendations for future research.

## Results

### Characteristics of the sequencing data

**Fig. 1:**
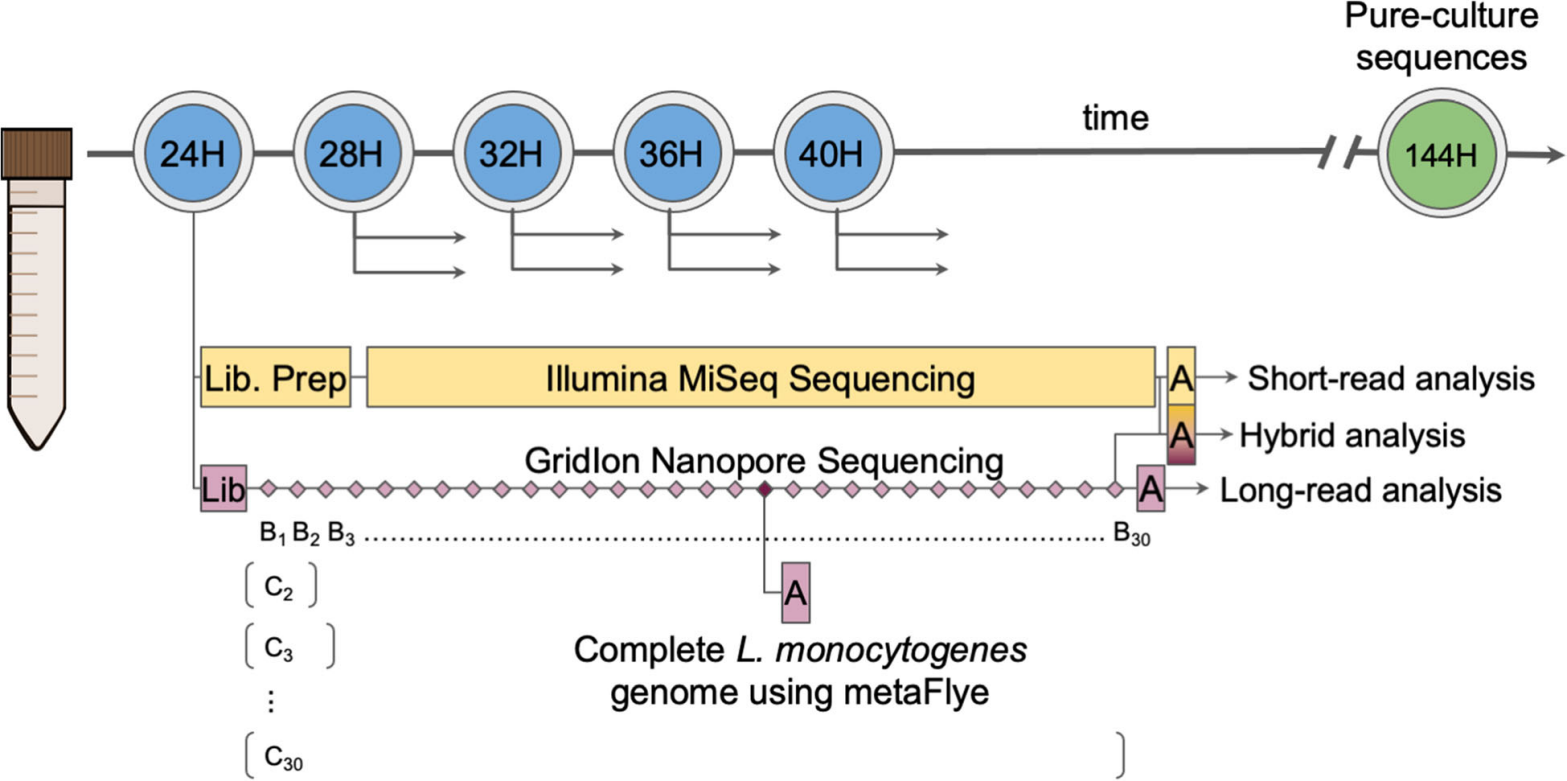
The effective time required to sequence and analyze the quasimetagenomic samples. The blue circles marked as 24H, 28H, 32H, 36H, and 40H denote the five enrichment time points where the quasimetagenomic samples were collected and sequenced with the Illumina MiSeq (short read) and the Oxford Nanopore GridIon (long read). Diamonds represent the 30 batches (B_1_ to B_30_) of 4000 GridIon reads, each generated 45 min apart. For our analysis, reads from each batch were merged with previously obtained batches to form cumulative batches (C_i_). The time taken to assemble the reads is shown with boxes labeled ‘A’. C_18_ at 24H marks the earliest time point where a complete *Listeria monocytogenes* genome was reconstructed (with metaFlye). The green circle corresponds to the time required to culture and sequence a pure colony isolate of *Listeria monocytogenes* i.e. 144 h. *Note: bioinformatic analysis can be performed in “real-time” on the GridIon batches as they are output whereas an Illumina MiSeq sequencing run must finish before the bioinformatics can begin. However, for our analysis we partitioned the reads from each MiSeq run into 30 batches—each composed of an equal number of sequenced bases as the GridIon batches*

The GridIon nanopore instrument generates sequencing data in batches of 4000 reads, denoted here as B_n_ for the n^th^ batch. The first 30 batches of GridIon reads, at each enrichment time, were used for this study, i.e., the first 120,000 reads corresponding to batches B_1_, B_2_, …,B_30_ (Fig. 1). To analyze the quality of assemblies as a function of increased sequencing depth, each successive batch of reads was combined with the previous batches for assembly to form “cumulative batches”, denoted as C_1_, C_2_,...,C_30_, where C_n_ = B_1_ + B_2_ + ... + B_n_ (Fig. 1). To compare assembly results strictly based on sequencing technology, the number of base pairs for the MiSeq and GridIon data was normalized. Over a range of sequencing depths, MiSeq raw read files were partitioned into 30 corresponding batches of read pairs to match the cumulative batches by number of base pairs for GridIon reads. Table 1 records the total number of sequenced bases per C_30_ at each enrichment time.

**Table 1:** Summary of sequence data for C_30_ at each enrichment time

	24H	28H	32H	36H	40H
Sequenced base pairs	2.3 × 10^8^	3.3 × 10^8^	3.9 × 10^8^	5.4 × 10^8^	5.0 × 10^8^
Number of GridIon reads	1.2 × 10^5^	1.2 × 10^5^	1.2 × 10^5^	1.2 × 10^5^	1.2 × 10^5^
MiSeq reads in C_30_ (total MiSeq reads sequenced)	1.2 × 10^6^ (2.9 × 10^6^)	1.9 × 10^6^ (4.0 × 10^6^)	2.2 × 10^6^ (3.6 × 10^6^)	3.0 × 10^6^ (3.5 × 10^6^)	2.7 × 10^6^ (2.9 × 10^6^)

The mean read length for C_30_ across enrichment time points ranged from 174 to 198 nucleotides for Illumina MiSeq and 1923 to 4445 nucleotides for Oxford Nanopore GridIon. The longest sequenced GridIon read was 69,402 nucleotides long (Table 2). For the GridIon, there was a general increase in the mean and maximum read length as the enrichment time increased. Furthermore, the reads that mapped to the *L. monocytogenes* reference genome had a longer mean and maximum length compared to the rest of the reads across all enrichment time points (Supplementary Figure 1). The putative *L. monocytogenes* reads also had a much lower mean GC content (38%) compared to the rest of the reads (49–54%) across enrichment time points (Supplementary Figure 2).

**Table 2:** GridIon read length and sequencing error statistics for C_30_

Enrichment time (hours)	Mean read length	Max. read length	Average quality score	Min. est. sequencing error rate	Max. est. sequencing error rate
24	1923	48,588	21.8	7%	18%
28	2721	55,258	22.9	6%	17%
32	3268	57,233	22.8	7%	16%
36	4445	62,426	23.2	6%	13%
40	4129	69,402	23.2	6%	13%

The sequencing error rate for the reads mapping to the *L. monocytogenes* reference genome was 0.03% for the MiSeq reads and between 6.3 and 18% for the GridIon reads. The GridIon sequencing error rate has a range based upon whether the soft-clipping of read alignments (i.e. the ends of the reads not included in the alignment range) was included as error or not. Each read is thus assigned two error estimates: an upper estimate of error that treats the unaligned portion of the read as an error, and a lower estimate that relies solely on the errors identified within the aligned range. Insertions, deletions, and mismatches were only counted for the aligned portion of the reads i.e. excluding the soft-clipped regions. For the long reads, 29.6%, 25.4%, and 45% of the errors were due to mismatches, insertions, and deletions, respectively—in accordance with previously published results [14]. For the MiSeq, the sequencing error rate and mean base quality were relatively uniform across samples. For the GridIon, the estimated sequencing error rate range decreased from 24H (7% to 18%) to 40H (6.3% to 13%) while the mean per-base quality score slightly increased over the same time period, from 21.83 to 23.19, respectively.

### Selection of the reference genome

The accuracy of the assemblies was assessed with respect to a complete reference genome that had been isolated and sequenced (PacBio SMRT technology) from ice cream samples from the same facility as used for our analysis [37]. The reference was treated as a “gold standard” with an expected accuracy of ~ 99.999% [38]. Previous research had shown that the outbreak consisted of two strains. One that was only isolated from Facility 1 and another that was mainly isolated from Facility 2 [37]. The ice cream samples used for our analysis came from Facility 1. The reference genome used here had been used as a reference for SNP analysis of the isolates from Facility 1, showing they differed by 29 SNPs or fewer. Another reference genome, from Facility 2, had been used as the representative of the second strain. The C_30_ MegaHit quasimetagenome assemblies showed a higher similarity with the reference from Facility 1 than Facility 2 (mean Mash [39] distance: 0.0206 and 0.0218 respectively). The reference from Facility 1 was subsequently used for our analysis.

The similarity between the *L. monocytogenes* contigs derived from the quasimetagenomes and the reference sequence was assessed, and 55 loci were identified (46 single nucleotide insertions, 2 di-nucleotide insertions, and 5 single nucleotide polymorphisms) that differed at all enrichment times. Four of these variants (1 single nucleotide polymorphism and 3 single nucleotide insertions) occurred within the core of the *L. monocytogenes* genome (see Methods for a description of how the core was defined).

### Assessing the presence of multiple *L. monocytogenes strains*

The presence of multiple, closely-related *L. monocytogenes* strains in the quasimetagenomes could affect the accuracy of the assemblies. A prior analysis of the ice cream samples [20] had identified three putative co-occurring *L. monocytogenes* strains based upon the detection of three 16S rRNA gene variants. However, analysis of the 16S rRNA genes in the reference genome identified 6 copies of the 16S rRNA operon which clustered, by sequence, within three distinct clusters consistent with the originally-determined variants.

The presence of multiple strains in the quasimetagenomes was assessed and 586 loci were identified (75 within the core genes) where the pile-up of MiSeq reads indicated the presence of two alleles, i.e. the reference allele and a variant. The percent of reads supporting the variants had a normal distribution with a mean of 17% and a standard deviation of 4%—indicating a 5:1 ratio of relative abundance. This evidence suggests that two highly-clonal strains co-occur in our quasimetagenomic samples.

### General quasimetagenome assembly statistics

Ten assembly approaches were tested (Table 3), which were grouped into four broad categories: short read, long read, short read hybrid and long read hybrid. For simplicity, a tool was defined as a hybrid assembly approach if it used both short and long reads whether it be short read assemblies that get scaffolded with long reads (short read hybrid) or long read assemblies that get polished with short reads (long read hybrid).

**Table 3:** The ten assembly approaches tested

Tool	Application	Abbreviation
MegaHit	short read metagenome assembler	short read
Redbean	long read genome assembler	long read
Canu	long read genome assembler	long read
metaFlye	long read metagenome assembler	long read
Racon	polishing long read assemblies with long reads	long read
HybridSpades	hybrid genome assembler; short read assembly followed by long read scaffolding	short read hybrid
Opera-MS	hybrid metagenome assembler; short read assembly followed by long read scaffolding either (1) de novo or (2) using reference genomes	short read hybrid
ntEdit	polishing long read assemblies with short reads	long read hybrid
Pilon	polishing long read assemblies with short reads	long read hybrid

All assembly approaches had a mean runtime (for the full set of reads, C_30_, across enrichment times) of approximately 40 min or less (Table 4) except Canu which had a mean runtime of 98 min per sample. The fastest assembly approach was Redbean with a mean runtime of just one minute (Supplementary Figure 3).

**Table 4:** Mean assembly statistics (C_30_ at each enrichment time) for each assembly approach

Assembly tool	Runtime	Total assembly length	Number of contigs	N50	Longest contig
metaFlye (long read)	40.6	4,291,417	27	3,056,133	3,056,133
Canu (long read)	98	3,470,967	21	1,754,979	2,071,553
Redbean (long read)	1	3,474,503	35	2,123,769	2,131,343
MegaHit (short read)	32.8	7,972,605	7315	97,577	672,182
metaFlye+Racon (long read)	41.6	4,261,624	27	3,039,238	3,039,238
HybridSpades (short read hybrid)	22.6	11,681,048	19,285	112,850	686,270
OperaMS (no reference) (short read hybrid)	12.2	10,340,211	13,921	105,382	655,220
OperaMS (reference) (short read hybrid)	13.6	10,363,273	13,913	205,943	1,919,416
metaFlye+Racon+Pilon (long read hybrid)	41.6	4,271,759	27	3,041,086	3,041,086
metaFlye+Racon+ntEdit (long read hybrid)	41.6	4,274,358	27	3,041,440	3,041,440

The contiguity of the assemblies was measured using several metrics: the total assembly length (Supplementary Figure 4), number of contigs (Supplementary Figure 5), N50 (Supplementary Figure 6), and longest contig assembled (Supplementary Figure 7). The mean values for C_30_ across enrichment times for each contiguity metric are described in Table 4. Approaches that first assemble short reads (short read and short read hybrid assemblies) contrasted substantially with those that first assemble long reads (long read and long read hybrid assemblies) having consistently longer total assembly lengths, orders of magnitude more contigs, lower N50s, and shorter longest contigs. In general, as the enrichment of *L. monocytogenes* progressed, there was a general decrease in the number of contigs and total assembly size (Supplementary Figures 4 and 5).

As expected, the long read and long read hybrid assemblies had the highest N50 values and the longest contigs—often near the reference genome length for *L. monocytogenes* (~ 3 Mbp). Amongst the long read assembly tools, the metagenome assembler metaFlye consistently produced the highest N50 values with the longest contigs nearest to the length of the *L. monocytogenes* reference genome (Table 4); however, the differences between long read assembly tools decreased with enrichment.

In contrast, the short read and short read hybrid assemblies had low N50 values and the longest contigs assembled were consistently shorter (often by orders of magnitude) with little to no increase beyond 60X depth of coverage. Opera-MS, using reference-guided scaffolding, was the main exception, assembling contigs of 2 Mbp or more at all enrichment time points.

### Taxonomic composition of the quasimetagenomic samples

**Fig. 2:**
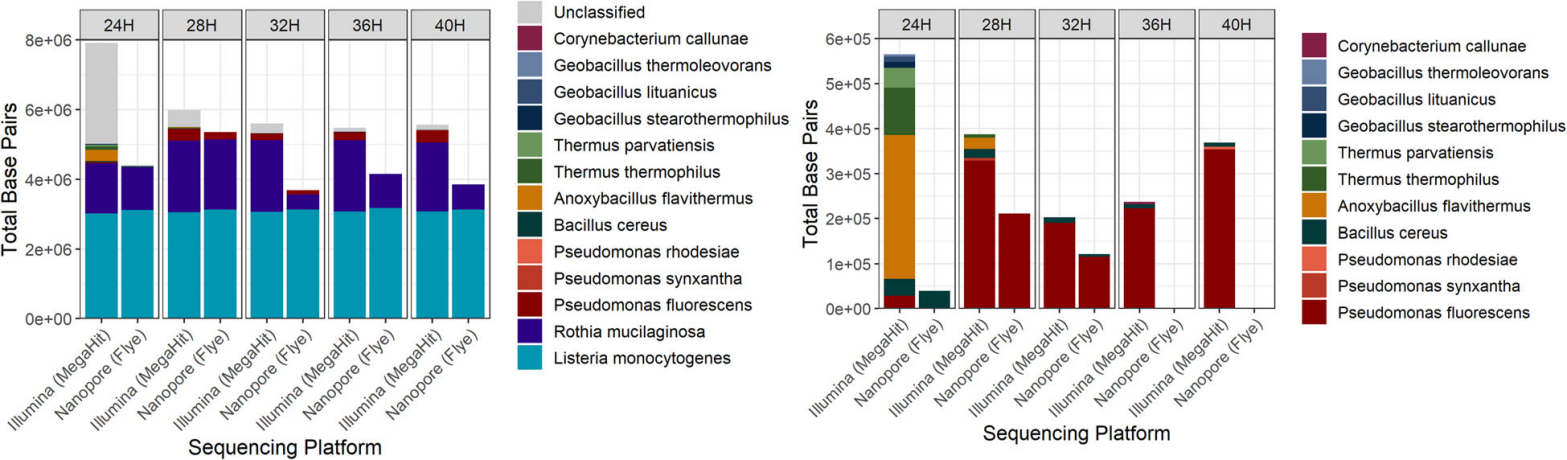
Taxonomic classification of cumulative batch 30 from each enrichment time point. For clarity, only the short read MegaHit and long read metaFlye assemblies were plotted (short read assembly results mirrored short read hybrid assemblies and long read assemblies mirrored long read hybrid assemblies). **a** The total bp of contigs per species (must have a minimum of 5000 bp) classified by Kraken. **b** Species in sample, excluding *L. monocytogenes*, *R. mucilaginosa* and unclassified sequences highlights how the short read assemblies capture more species than the long read assemblies

The number of species identified in the assemblies ranged from 2 to 10 with the short read and short read hybrid assemblies containing more species than the long read and long read hybrid assemblies (Fig. 2). The number of species decreased with enrichment time, and *L. monocytogenes* and *Rothia mucilaginosa* were the only species detected at all time points. *Bacillus cereus* was the most closely related species to *L. monocytogenes* detected in the quasimetagenomes (both species are members of the order *Bacillales*).

*L. monocytogenes* was the most abundant species at all times and its abundance increased with enrichment time, but the abundance estimates differed for the MiSeq and GridIon (Table 5). At 24H, 33% and 60% of the MiSeq and GridIon reads, respectively, mapped to the *L. monocytogenes* reference genome. At 40H, 92% and 97% of the MiSeq and GridIon reads respectively, mapped to the reference genome.

**Table 5:** Percent of reads that map to the *L. monocytogenes* reference genome

Enrichment time (hour)	MiSeq (reads mapped with Bowtie2)	GridIon (reads mapped with MiniMap2)
24	33	60
28	68	88
32	75	94
36	88	97
40	92	97

### Reconstruction of the *L. monocytogenes* genome from the quasimetagenomes

**Fig. 3:**
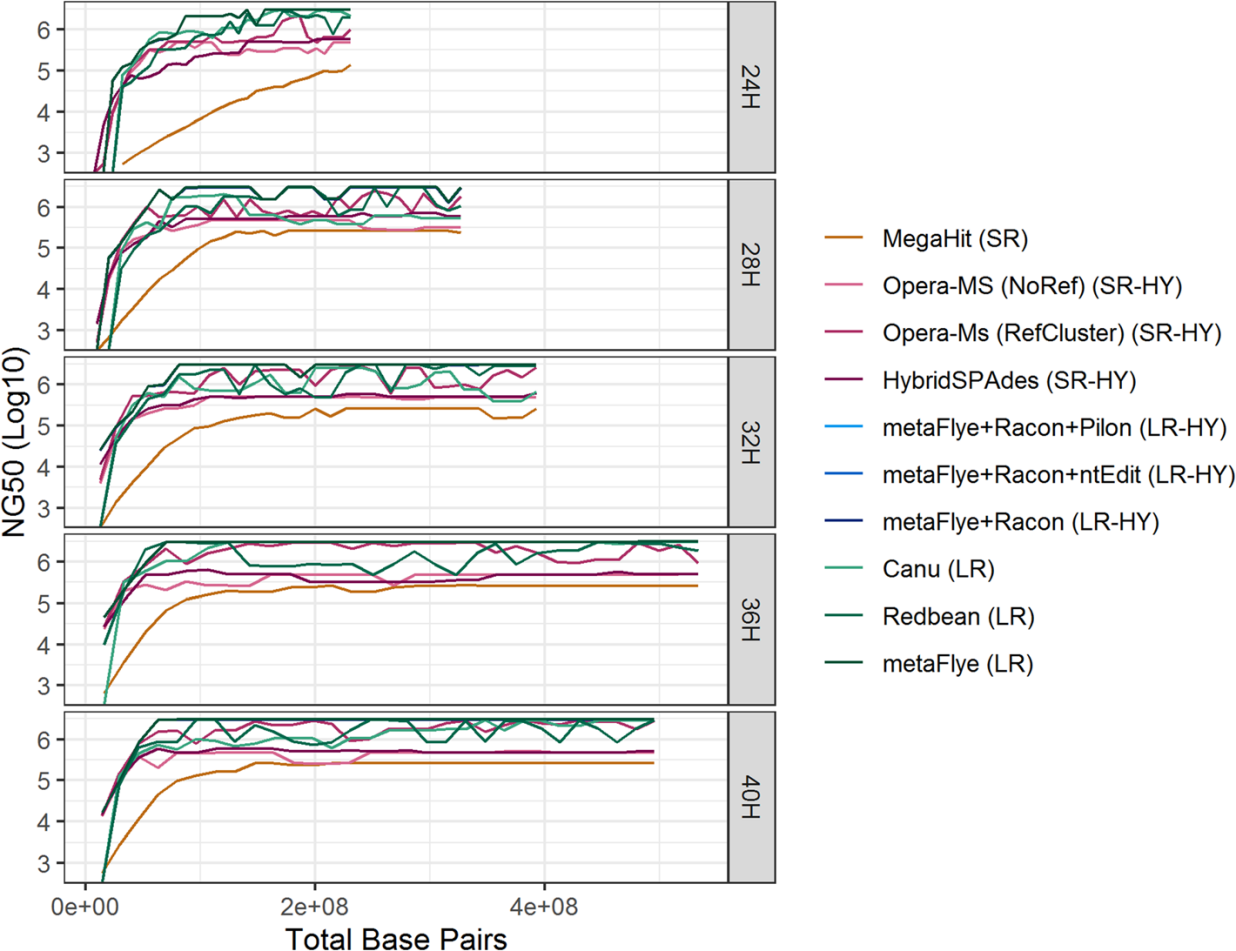
The NG50 versus the total number of base pairs sequenced per cumulative batch for the assembled *L. monocytogenes* contigs at each of the enrichment time points for each assembly approach. (Abbreviations: SR = short read, LR = long read, HY = hybrid)

The most contiguous recovery of the *L. monocytogenes* genome, as measured by the mean NG50 across enrichment time points (only using C_30_ at each time point), was by long read and long read hybrid assembly approaches (Fig. 3). For the long read assemblers Canu, Redbean, and metaFlye the mean NG50 values were 1,535,966 bp, 1,568,760 bp, and 2,490,733 bp, respectively. Because metaFlye assembled genome-length contigs for *L. monocytogenes* the most consistently of the long read assemblers, only the metaFlye assemblies were used for the long read hybrid assemblies. The long read hybrid approaches (using metaFlya and Racon in combination with Pilon or ntEdit) slightly decreased the mean NG50 of the metaFlye assemblies, 2,477,272 bp, 2,478,715 bp, 2,478,772 bp, respectively.

The short read Megahit assemblies had the smallest mean NG50 at 162,346 bp. The short read hybrid assemblies of HybridSpades and Opera-MS without reference-guided scaffolding had mean NG50’s that were several fold higher than the Megahit assemblies, 431,211 bp and 375,881 bp, respectively. Opera-MS, using reference-guided scaffolding, had a mean NG50 of 1,414,301 bp, nearly an order of magnitude higher than Megahit and close to that of the long read assembler Canu.

Only the long read assemblers were able to assemble genome-length contigs (over 3 million bp) for *L. monocytogenes*. The earliest complete reconstruction of the *L. monocytogenes* genome was at 24H and C_18_ with metaFlye (33X depth of coverage of the *L. monocytogenes* genome), 24H and C_22_ with Canu (40X depth of coverage of the *L. monocytogenes* genome), and 28H and C_16_ with Redbean (47X depth of coverage of the *L. monocytogenes* genome). The genome length contigs, irrespective of the long read assembly approach, were frequently up to tens of thousands of base pairs longer than the reference genome, mainly due to over-circularization of the assembly by a read length or less. Additionally, each long read assembler recovered a circularized 71 kbp putative *L. monocytogenes* plasmid that was always fragmented in the short read assemblies. The best BLAST hits within the NCBI nt database for the assembled plasmid were to known *L. monocytogenes* plasmids (NCBI accessions CP053631.1 and CP044431.1). The plasmid was not found to host any known resistance or virulence genes.

### Assembly errors in the *L. monocytogenes* genomes reconstructed from the quasimetagenomes

**Fig. 4:**
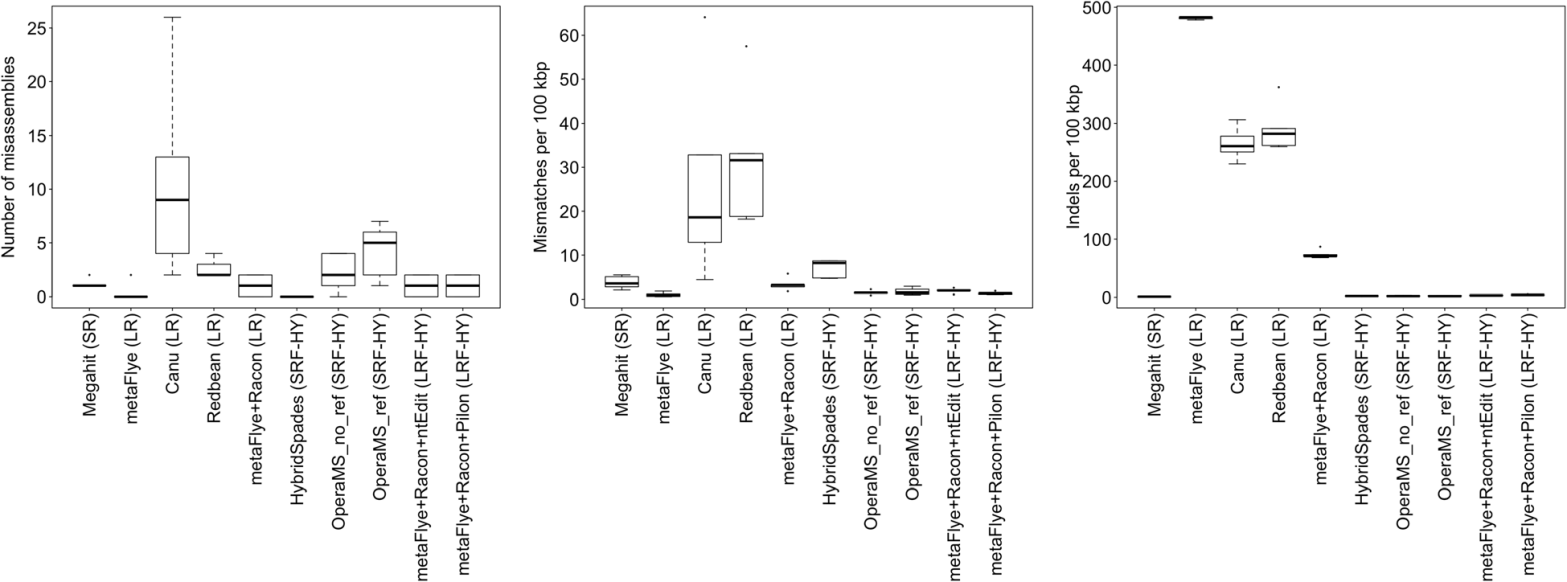
The quality of assembled contigs annotated as *L. monocytogenes*, with respect to the reference genome, using Quast for cumulative batch 30 at each of the enrichment time points. The number of mismatches, insertion/deletion (indels), and misassemblies per 100 kbp for each assembly approach. (Abbreviations: SR = short read, LR = long read, HY = hybrid)

Quast was used to compare the mean number of misassemblies, mismatches per 100 Kbp, and indels (insertions and deletions) per 100 Kbp in the *L. monocytogenes* contigs for each assembly approach, given the highest sequencing depth of coverage of the quasimetagenomes (i.e. C_30_) across enrichment times (Fig. 4). The number of misassemblies and mismatches varied more by tool than assembly strategy. The mean number of misassemblies ranged from 10.8 (Canu) to 0 (HybridSpades). The mean number of mismatches per 100 Kbp ranged from 31.8 (Redbean) to 1.2 (metaFlye). In contrast, the long read assembly approaches had a pronounced indel rate versus other approaches, ranging from 265 (Canu) to 481 (metaFlye). The combination of metaFlye with Racon substantially reduced the number of indels to 74 per 100 kbp. Combining short read and long read information with long read hybrid assembly approaches further reduced the number of indels to ~ 3 per 100 kbp. Short read assembly/short read hybrid assembly approaches had the lowest indel rate of around 1 to 2 per 100 kbp.

### Accuracy of the *L. monocytogenes* metagenome-assembled genomes

At all enrichment time points and C_30_ reads (for both short and long reads), there was 100% breadth of coverage of the *L. monocytogenes* reference genome and up to ~160X depth of coverage.

The fraction of the *L. monocytogenes* genome that was typeable by the MiSeq and GridIon reads was assessed by identifying regions in the reference genome where the C_30_ reads mapped ambiguously (i.e. mapped with the same alignment score to multiple genome locations). For the MiSeq and GridIon reads, a median of 3.9% (118,615 bp) and 0% (0 bp), respectively, of the reference genome consisted of ambiguous regions.

Earlier results provided evidence for the presence (with a 5:1 relative abundance ratio) of two strains of *L. monocytogenes* in the quasimetagenomes. The less abundant strain differed from the more abundant strain at 586 loci. Analysis with Snippy showed that no more than 13 of the 586 variants in the low abundance strain were present in a given C_30_ assembly (across enrichment times). However, the long read assemblies contained the highest median number of variants (maximum was 12 with metaFlye) while the other assembly approaches had a median of 3 or less.

**Fig. 5:**
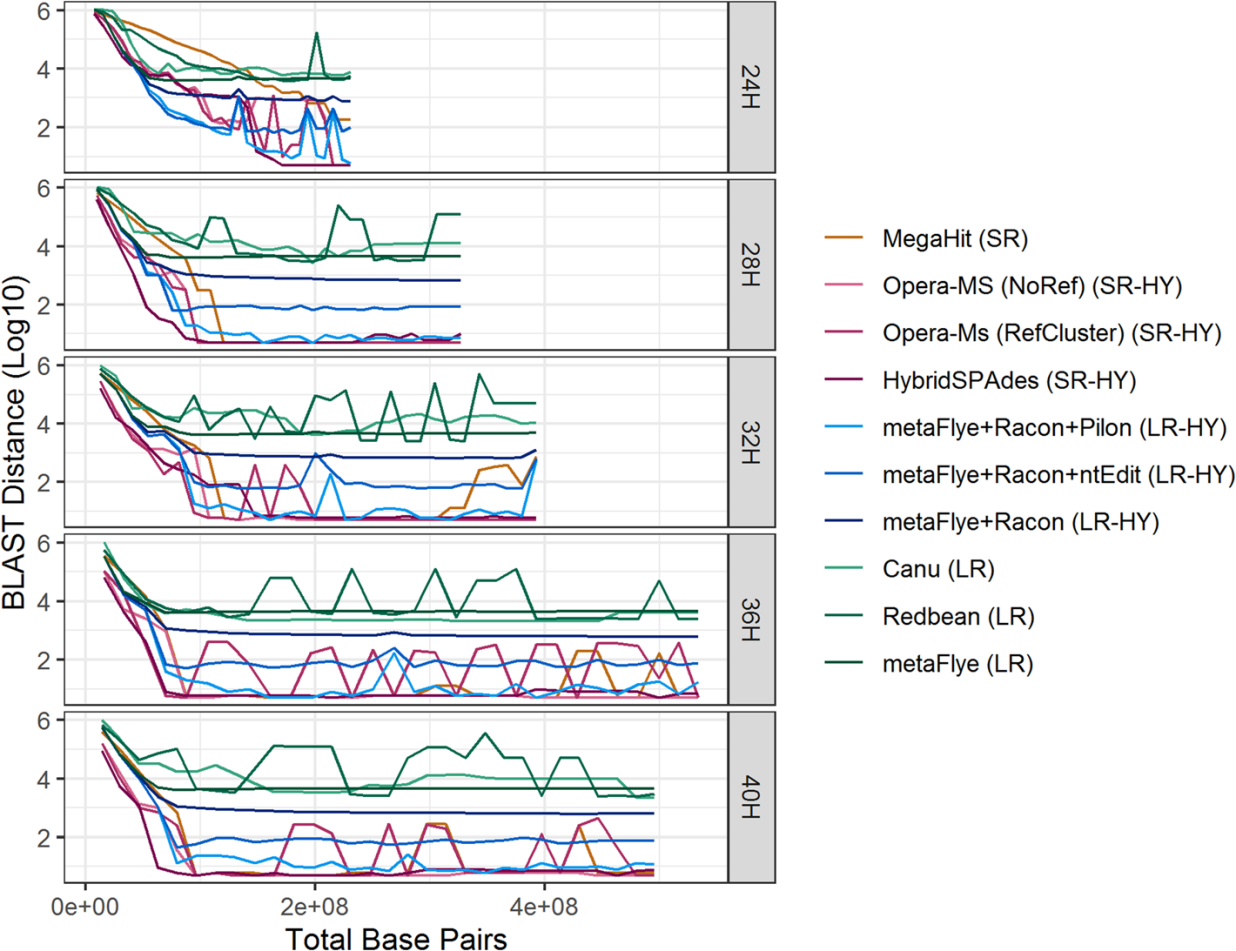
Core gene BLAST distances. BLAST distance between the core genes of the reference genome and the assemblies versus the total number of base pairs sequenced per cumulative batch. (Abbreviations: SR = short read, LR = long read, HY = hybrid)

**Fig. 6:**
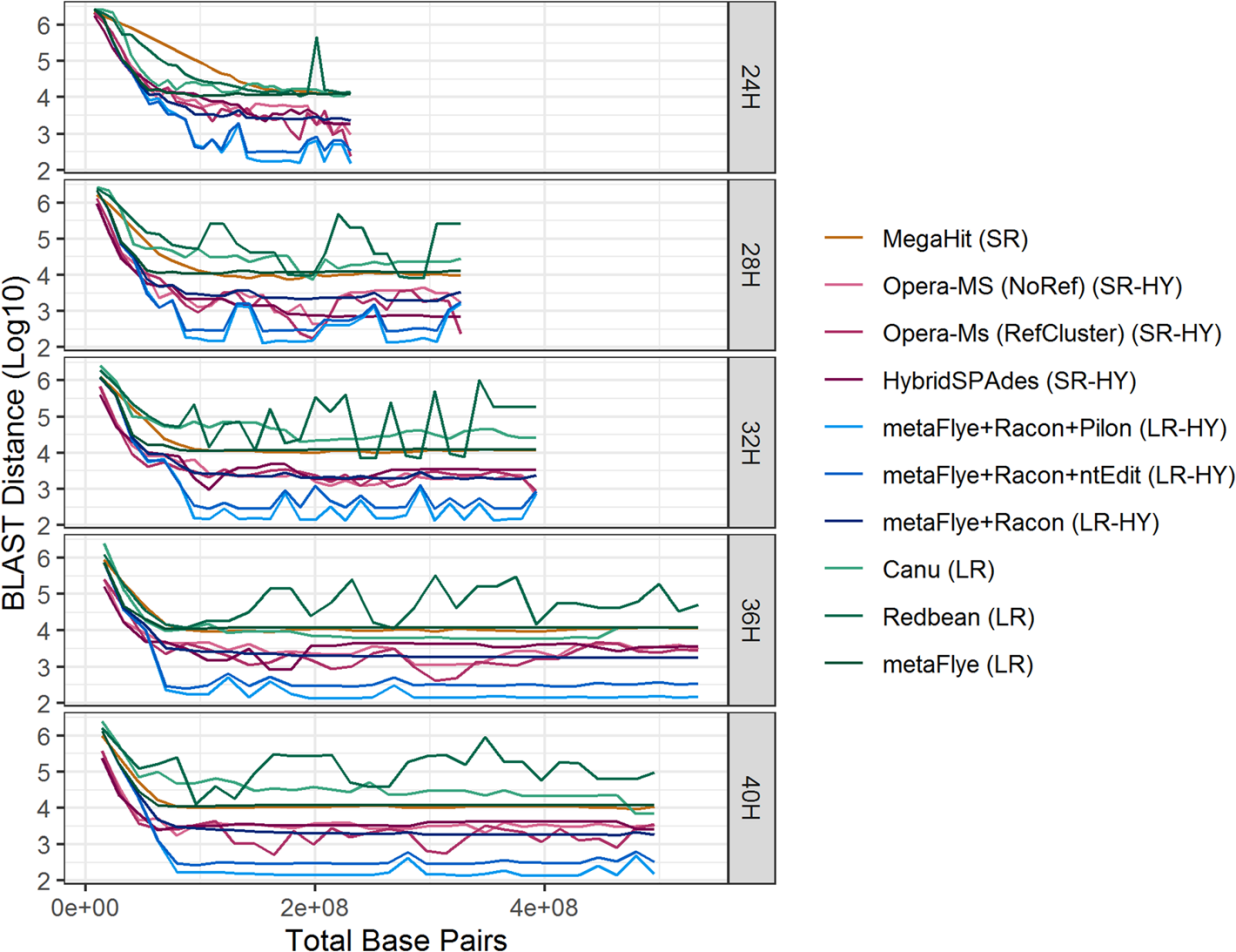
Complete gene set BLAST distances. BLAST distance between the complete gene set of the reference genome and the assemblies versus the total number of base pairs sequenced per cumulative batch. (Abbreviations: SR = short read, LR = long read, HY = hybrid)

Next, the accuracy of the assemblies (C_1_ to C_30_ at each enrichment time) was assessed by calculating the BLAST distance between the core genes (Fig. 5) and the complete set of genes (Fig. 6) of the reference genome and the *L. monocytogenes* contigs. As defined earlier, the BLAST distance is a measure of sequence similarity equalling the number of mismatches, insertions, and deletions in the BLAST alignment between the reference genes and the assembled genes. The short read and short read hybrid assemblies attained the smallest BLAST distances for the core genes, while the long read hybrid assemblies attained the smallest BLAST distances for the complete set of genes.

For the core genes, the smallest BLAST distance observed was 5 (Fig. 5). Four of the differences were caused by variants identified previously in the core genes of the *L. monocytogenes* extracted from the quasimetagenomes. The fifth difference varied in location for different assemblies, and showed no relation to the variants discovered previously.

The short read hybrid approaches assembled the core genes with BLAST distance 5 at the earliest time point: HybridSpades at 24H and C_22_ corresponding to 40X (long reads) and 19X (short reads) depth of coverage of the *L. monocytogenes* reference genome; Opera-MS, both with and without reference-guided scaffolding, at 24H and C_28_ corresponding to 50X (long reads) and 25X (short reads) depth of coverage of the *L. monocytogenes* reference genome. Megahit assemblies attained a BLAST distance of 5 after 28H and C_11_ corresponding to 28X depth of coverage of the *L. monocytogenes* reference genome. At 24H, 28H and 36H the short read hybrid assemblies obtained a BLAST distance 5 with fewer short reads than the short read assemblies; however, at 32H and 40H, the short read and short read hybrid assemblies required the same amount of short read data to achieve a BLAST distance of 5.

The long read assemblies never achieved a BLAST distance of less than 2000 even with 158X depth of coverage of *L. monocytogenes*. Polishing the long read metaFlye assemblies with Racon improved the assembly of the core genes, achieving a minimum BLAST distance of 609. Long read hybrid assembly with Pilon achieved a BLAST distance of 5 at 28H and C_14_ which corresponded to 36X (short reads) and 38X (long reads) depth of coverage of the *L. monocytogenes* reference genome; however, it achieved BLAST distance 5 less consistently than short read or short read hybrid approaches (Fig. 5). Long read hybrid assembly with ntEdit assembled the core genes with less accuracy than Pilon, with a median BLAST distance (C_1_ to C_30_ across enrichment times) of 81 and 11, respectively.

The long read hybrid approaches assembled the complete gene set with the lowest BLAST distance, with Pilon outperforming ntEdit (Fig. 6). Pilon achieved a BLAST distance of 132, the best observed for any tool, at 28H and C_14_ corresponding to 36X (short reads) and 38X (long reads) depth of coverage of the *L. monocytogenes* reference genome. The mean BLAST distance across enrichment time points was 699 for Pilon and 798 for ntEdit. None of the other assembly approaches attained this level of accuracy. For reference, the next best tool, metaFlye+Racon, had a mean BLAST distance of 2991.

### Variation in assembly quality between successive cumulative batches

**Fig. 7:**
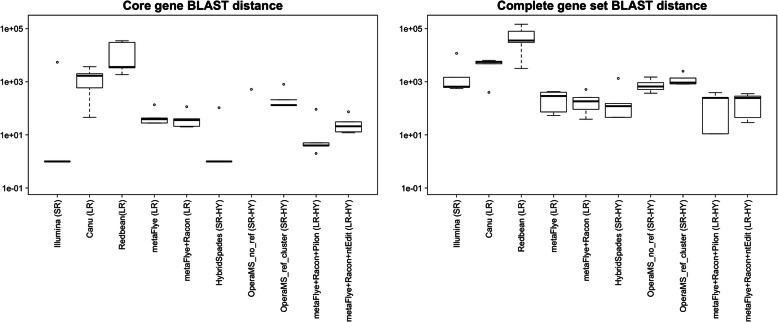
Consistency of assembly approaches between successive cumulative batches. Median successive cumulative batch difference in BLAST distances, across enrichment time points, for the A) the core genes and B) the complete gene

In addition to accuracy, the precision with which assemblies can be reconstructed is of great importance for pathogen detection. The accuracy of the assembly approaches (in terms of divergence in core and full gene sets with respect to the *L. monocytogenes* reference) varied widely between successive cumulative batches (Fig. 7).

The tools that most consistently assembled the core genes were Opera-MS (without reference guided scaffolding), HybridSpades, MegaHit, and metaFlye+Racon+Pilon; the median difference in BLAST distance between successive cumulative batches for these tools was 0, 1, 1, and 5, respectively, across all enrichment time points. All assembly approaches had a median difference in BLAST distance of less than 50 except Opera-MS with reference guided scaffolding (121), Canu (1183) and Redbean (10,930).

The variability in the accuracy for the reconstruction of the complete gene set was an order of magnitude greater than for the core genes. The most consistent tools were HybridSpades, metaFlye+Racon+Pilon, metaFlye+Racon+ntEdit, metaFlye+Racon, and metaFlye—the median difference in BLAST distance between cumulative batches was 132, 137, 140, 183, and 207, respectively. All assembly approaches had a median difference in BLAST distance of less than 1000, with the exceptions of Opera-MS with reference guided scaffolding (1019), Canu (4758), and Redbean (32,655).

### Depth of coverage did not always improve assembly quality

Increased depth of coverage did not always correlate with improved performance in assembly metrics. For example, the longest contig assembled by the short read assemblies was very similar at 30X depth of coverage and at 150X depth of coverage, 695,760 nt and 695,778 nt, respectively. In some cases, the performance of assembly approaches actually decreased with increased depth of coverage. For example, the lowest BLAST distance for the complete gene set for the metaFlye+Racon+Pilon assemblies increased from 132 to 153 despite an increase of 100X depth of coverage of the *L. monocytogenes* genome for both short and long reads.

## Discussion

Public health labs are continually developing and testing new methods and approaches to increase the speed and resolution of pathogen source tracking. Expediting source attribution will contribute to reduced illnesses, deaths and the economic burden of illness outbreaks. Currently, the standard workflow for strain typing and source attribution involves sequencing genomes (primarily with Illumina MiSeq technology) of isolated colonies, cultured from selective enrichments. Sequence data is analyzed using SNP and/or MLST analyses. Here we evaluated the contribution of quasimetagenomics and the applied integration of (short) MiSeq and (long) GridIon reads for the improvement of this workflow.

### Quasimetagenomics expedites source tracking

Currently, direct metagenomic sequencing of samples cannot replace genome sequencing of culture isolates for the strain typing of pathogens; however, quasimetagenomics has shown great promise for reducing the amount of enrichment time needed to type pathogens with sequence data [26, 35, 36]. Previous work on the listeriosis ice cream outbreak demonstrated that quasimetagenomic short read sequencing provided sufficient coverage of the *L. monocytogenes* genome to determine its membership in the outbreak cluster at 24 h enrichment—a significant improvement over the ~ 6 day procedure required to culture and sequence an isolate genome [26]. This work supports that MiSeq short read sequencing can expedite the recovery of a target pathogen from quasimetagenomes, accurately reconstructing the *L. monocytogenes* core genes at 28 h of enrichment. Further, the integration of MiSeq short read and GridIon long read sequencing further expedited the accurate assembly of the core genes and increased the contiguity of assemblies—including the reconstruction of a complete genome and plasmid—at 24 h of enrichment (Fig. 1). This highlights that an integrated approach to quasimetagenomics can greatly expedite and enhance source tracking.

### Long reads have added value over short reads for quasimetagenomics

Although short reads can be used for high resolution SNP and cgMLST/wgMLST analyses they cannot span many genomic repeat regions, resulting in fragmented assemblies that preclude the recovery of complete bacterial genomes [13]. The fragmented assemblies can prevent the identification of genes, gene synteny, repeats, structural variants, and extrachromosomal sequences, like plasmids and phages, that could be readily observed in complete assemblies.

Our results showed that ~ 4% of the *L. monocytogenes* genome was not typeable by the MiSeq reads. In contrast, the entire *L. monocytogenes* genome was typeable with the GridIon reads, enabling the complete reconstruction of the *L. monocytogenes* genome and plasmid at 24 h of enrichment and only 33X depth of coverage. The ability of long reads to span genomic repeats will support much higher resolution whole genome based source tracking methods and provide detailed information about the mobileome. However, similar to previous studies [40], we found the high sequencing error rate of the nanopore reads to induce incorrect base calls in the assembled sequence, thereby negatively impacting strain typing and strain attribution. Nonetheless, with time, we expect the sequencing error rate to decrease and the utility of nanopore sequencing for source tracking to increase substantially.

Another advantage of nanopore over MiSeq sequencing is that the data is output in batches of reads every 30 to 60 min (as opposed to a MiSeq sequencing run which takes ~ 24 h depending on the number of cycles). As assembling the reads is much faster than sequencing itself (Fig. 1), nanopore sequencing allows the analysis to terminate as soon as sufficient reads have been obtained for accurate analysis—a point that may vary depending on the characteristics of the sample. This ability can greatly expedite source tracking by facilitating near-real-time bioinformatic analyses.

### Hybrid assembly outperforms other approaches but with trade-offs

Our results support the accuracy of hybrid assemblies [17, 18, 36]—hybrid assembly, using both Illumina short reads and nanopore long reads, could reconstruct more complete and accurate genomes than using either of the platforms alone. However, the initial assembly strategy (i.e. whether the short reads were assembled first or long reads) had a substantial impact on the quality of the reconstructed genomes. Short read hybrid assembly approaches led to a more accurate assembly of the core genes, but the assemblies were more fragmented. The use of reference genomes to scaffold assemblies increased the contiguity of the short read hybrid assemblies, but also introduced assembly errors—a potential consequence if the references used for scaffolding has structural differences compared with the genomes being assembled. For the long read hybrid assembly approaches, a higher indel rate prevented the accurate assembly of the core genes; however, the assemblies had higher contiguity, sometimes reconstructing the complete *L. monocytogenes* genome. Additionally, the long read hybrid assembly approaches led to the most accurate recovery of the complete set of genes, with potential implications for characterizing the phenotype (e.g., drug resistance) of the pathogen. The choice of the hybrid assembly approach can be made subject to whether the application of the reconstructed genome mandates highly accurate core genes or an overall accurate complete genome.

### Short read based assembly approaches showed the best performance

Assemblies need to be accurately reconstructed to be useful for SNP and cgMLST/wgMLST based source tracking analyses. Among the assembly approaches tested, the most accurate was the reconstruction of the core genes using either the short read or short read hybrid assembly strategy. Short read hybrid assembly was consistently able to accurately assemble the core genes with the same amount or fewer short reads than the short read assemblies. However, the combined use of short and long reads entails higher costs in both personnel time and reagents, which may not be justified as similar accuracy can be obtained with short reads alone at a slightly higher depth of coverage. In contrast, no assembly approach could reconstruct the complete set of genes with high accuracy or consistency, although long read hybrid approaches were by far the best performing. Nonetheless, given a lower sequencing error rate, long read approaches might become preferrable with the added value of assembling complete genomes and mobile elements like plasmids.

### Areas of improvement for assembly algorithms

At the time of our analysis, metaFlye was the only metagenomic long read assembler available, and it performed better than the other long read assemblers in our application. This observation highlights that long read assemblers developed for single genomes are not effective when samples contain mixtures of DNA from multiple organisms—suggesting the need for further research in developing efficient metagenomic assembly tools for long read data. Additionally, the quality of the metaFlye assemblies was improved considerably by polishing the assemblies with the long reads themselves, indicating that none of the long read assemblers make full use of the information available in the long reads.

The observed differences between hybrid assembly approaches that start with short reads and those that start with long reads, suggest that hybrid approaches are currently limited by the weaknesses of the different technologies. This highlights the scope for improvement in hybrid assembly approaches, underscoring that we are still far from developing techniques which effectively integrate their strengths (e.g., the contiguity of long reads and high per-base quality of the short reads).

A weakness common to all assembly approaches was sensitivity to the addition of cumulative batches of sequence data, resulting in inconsistent gains/losses in assembly quality. This affected many metrics such as the N50 and the accuracy of the assembled genes. The differential sensitivity of assembly approaches to the addition of sequence data from the same sample suggests that assembly tools can be made more robust and consistent—greatly benefiting many applications including strain typing.

An advantage for quasimetagenomics is the detection of co-occurring strains that might be missed by traditional methods (i.e. culturing and sequencing a single isolate). Our analysis suggested the presence of at least two strains of *L. monocytogenes* in the quasimetagenomes. However, the current tools do not account for the variations within the (quasi-)metagenomic samples and current assembly approaches simply reconstruct the most abundant strain, which is what we observed with our assemblies. While further analysis of the data can reveal the strain structure hidden by the consensus assembly, we believe it is preferable that assemblers themselves account for and reveal the strains contained in the sample, information that could be valuable for source tracking.

## Conclusion

The integration of nanopore long read and Illumina short read sequencing expedited the reconstruction of high quality *L. monoctyogenes* assemblies from ice cream quasimetagenomes. The core genes were accurately reconstructed after 24 h enrichment with the short read hybrid assemblies and 28 h for the short read assemblies–a significant reduction from the standard 6 day protocol. Although the GridIon long read assemblies had too many errors to reconstruct the core genes with high fidelity, they had added value for reconstructing complete genomes and plasmids–providing information about synteny, gene content and genome structure that were not accessible with short reads. Hybrid assembly showed the best performance but with different weaknesses depending on whether the short or long reads built the initial assembly–highlighting areas for algorithmic improvement that integrate the strengths of long and short reads (e.g., the contiguity of long reads and high per-base quality of the short reads). A new and more complete level of information about genome structure, gene order and mobile elements can be added to the public health response by integrating microbiological (quasimetagenomic), molecular (long and short read sequencing) and optimized bioinformatic approaches.

## Methods

### Experimental design

Using long and short read sequencing technologies, we compared the performance of various assembly approaches for reconstructing the genome of *L. monocytogenes* from selective enrichments of naturally contaminated ice cream samples (Fig. 1). The isolation of a pure colony of *L. monocytogenes* for sequencing typically requires up to 6 days of selective culture enrichment [26]. During the selective enrichment, aliquots were collected at 4-h intervals from 24 to 40 h (denoted as 24H, 28H, 32H, 36H, 40H). MiSeq short read and GridIon long read sequencing were performed on DNA from these incremental enrichments. At each time point, over a range of sequenced depth of coverage of the quasimetagenomes, the sequence data was assembled using the short and long reads in combination and separately. Assembly quality was evaluated by comparison to a complete *L. monocytogenes* reference genome—sequenced and assembled from PacBio data—obtained from the full 6-day enrichment protocol.

### Enrichment

Ice cream samples, associated with the 2015 Blue Bell multistate listeriosis outbreak, were homogenized and added to Buffered *Listeria* Enrichment Broth (BLEB) with pyruvate according to the specifications outlined in Chapter 10 of the FDA BAM [19]. The mean MPN/g of *L. monocytogenes* in the ice cream samples was 11.99. After four hours, three filter sterilized selective agents (M52) were added to achieve final concentrations of 10 mg/L acriflavin, 40 mg/L cycloheximide, and 50 mg/L sodium nalidixic acid in the BLEB. Four replicates of negative (no ice-cream) and positive controls (*L. monocytogenes* cells) were also evaluated for bacterial growth every four hours over the 40-h enrichment.

### DNA extraction and sequencing for short reads

For each of the enrichment time points (24H, 28H, 32H, 36H, and 40H), DNA was extracted using DNeasy Blood and Tissue kit (Qiagen) following the protocol for Gram-positive bacteria with minor modifications: 1.5 ml of the culture was pelleted (5000×g, 15 min) and the pellet resuspended in 200 mL of enzymatic lysis buffer containing 20 mM Tris-HCl (pH -8.0), 2 mM Sodium EDTA, 1.2% Triton X- 100, 20 mg/ml of lysozyme. Samples were incubated for 60 min at 37 °C. Short read libraries were prepared with Nextera Flex (Illumina) library prep kit according to the manufacturer’s specifications. Libraries from enrichment time points 24H, 28H, 32H, 36H, and 40H were multiplexed along with 20 other libraries from different time points from the same study on to Illumina MiSeq 2 × 250 cartridge (Illumina, CA) following manufacturer recommended protocol.

### DNA extraction and sequencing for long reads

For each enrichment time point (24H, 28H, 32H, 36H, 40H), 2 ml aliquots of enrichment were removed and pelletized using a benchtop Centrifuge (Eppendorf 5418 R, NY, USA) at 4000 rpm for 10 mins. The pellet was resuspended in 300ul of TE Buffer. 300ul of the resuspended cells were loaded on the Maxwell® RSC Instrument (automated DNA extraction instrument, Madison, WI, USA) cartridge for DNA extraction. Genomic DNA was extracted using Maxwell® RSC Cultured Cells DNA Kit (Cat no: AS1260, Madison, WI, USA) on Maxwell RSC instrument following the manufacturer recommended protocol for Gram-positive bacteria.

Sequencing libraries were prepared using the ligation sequencing kit (Cat no: SQK-LSK109, Oxford Nanopore, Oxford, UK), according to the manufacturer’s specifications along with Native Barcoding Expansion 1–12 (Cat no: EXP-NBD104, Oxford Nanopore, Oxford, UK) for multiplexing the samples. The libraries were multiplexed into 2 pools (Pool1: 24H, 28H, 32H): Pool 2: 36H, 40H). The libraries were sequenced using GridIon with Flow cell (Cat no: FLO-MIN106, Oxford, UK) following the manufacturer’s recommended protocol. The GridIon outputs the raw signal data in batches of 4000 sequenced reads in fast5 format files [41]. Each fast5 file was converted into fastq formatted DNA sequences using Guppy for basecalling [42]. The fast base calling mode was used, which has a speed of ~ 4.6 Mbp/second. The GridIon typically outputs a batch of reads every 30 to 60 min (internal to lab), but is affected by factors such as the length and quality of the DNA fragments being sequenced.

### *L. monocytogenes* reference genome

Previous work identified two strains from the *L. monocytogenes* ice cream outbreak [37]. Two reference genomes (NZ_CP016213.1 and NZ_MAGN00000000.1) were used for the SNP analysis of the two strains. These reference genomes were compared with the *L. monocytogenes* assemblies from the quasimetagenomes using Mash (v2.0, k = 25, s = 100,000). The complete *L. monocytogenes* genome (Genbank accession NZ_CP016213.1) was more similar to the data in the quasimetagenomes (see Results section) and was used as the reference for our analyses. This reference organism had previously been isolated from a single colony at the end of the enrichment protocol and sequenced with PacBio RSII from ice cream samples from Facility 1, the same facility our samples came from [37]. The reference is 3,030,827 bp long with 2984 protein-coding genes (2,710,041 bp in total length) predicted by Prokka (v1.12) [43] and a GC-content of 38%. The core genes (using the 1013 gene cgMLST scheme developed for *L. monocytogenes* at the FDA [6, 7]) were identified by BLAST [44] alignment. The total length of the core genes was 1,075,554 bp. Six copies of the 16S rRNA were identified in the reference genome with BLAST using the RNAmmer database [45].

### Partitioning the sequenced reads into cumulative batches

The GridIon nanopore sequencing instrument generates the data in batches of 4000 reads, denoted here as B_n_ for the n^th^ batch. Our analysis used the first 30 batches of reads, i.e., the first 120,000 reads corresponding to batches B_1_, B_2_, …,B_30_ (Fig. 1). To analyze the quality of assemblies as a function of increased sequencing depth, each successive batch of reads was combined with the previous batches for assembly to form “cumulative batches”, denoted as C_1_, C_2_,...,C_30_, where C_n_ = B_1_ + B_2_ + ... + B_n_ (Fig. 1). To compare assembly results strictly based on sequencing technology, the number of base pairs for the MiSeq and GridIon data was normalized. Over a range of sequencing depths, MiSeq raw read files were partitioned into 30 corresponding batches of read pairs to match the cumulative batches by number of base pairs for GridIon reads. Table 1 records the total number of sequenced bases per C_30_ at each enrichment time.

### Detection of genomic variants and the presence of multiple strains

The detection of variants between the reference and the *L. monocytogenes* sequences reconstructed from the quasimetagenomes was conducted with two methods. In both cases, the MiSeq reads from cumulative batch C_30_ from each enrichment time were analyzed. The first method called variants with Snippy (v4.6) [46] if there was ≥10X depth of coverage and ≥ 95% of the reads supported the variant. The second method consisted of mapping the MiSeq reads to the reference genome with Bowtie2 (v2.3.4) [47] and analyzing the pile-up of reads with SAMtools (v1.7) [48]. Loci with ≥50X depth of coverage and where 20 to 90% of the aligned reads indicated the presence of another allele (while the rest of the aligned reads supported the reference allele) were considered to be evidence for multiple strains.

### Raw read statistics and reference genome coverage

Raw read statistics were collected for the 30 batches of reads (B_1_ to B_30_) per enrichment time point, including: mean per base quality score, number of reads, number of base pairs, read length distribution, and estimated sequencing error rate. To estimate the sequencing error rate, the short and long reads were mapped to the *L. monocytogenes* reference genome with Bowtie2 (v2.3.0) and MiniMap2 (v2.17-r974-dirty) [49], respectively, using default settings. The number of mismatches, insertions, and deletions were counted for the mapped reads with respect to the reference genome. For the GridIon reads, an estimated range was provided for the sequencing error rate because MiniMap2 is a local, as opposed to a global, read alignment tool. The range is based on whether soft-clipping of the read alignments is included as sequencing error (maximum estimate of error) or not (minimum estimate of error). Insertions, deletions, and mismatches were only counted for the aligned portion of the reads i.e. excluding the soft-clipped regions. The read mappings were used to estimate the breadth and depth of coverage (DOC) of the *L. monocytogenes* reference genome.

### Assembling the sequenced reads

Short reads and long-reads from each cumulative batch (C_1_ to C_30_) were assembled per enrichment time point (Fig. 1). The short reads were assembled using MegaHit (v1.2.9) [50] with default settings and scaffolded with MetaCarvel [51]. The long reads were assembled using Canu (v1.7) [52], Redbean (v2.5) [53], and metaFlye (v2.6-release) [54] with default settings. The Redbean assemblies were polished with MiniMap2 (v2.17-r974-dirty) and SAMtools (v1.5) following the tutorial for Redbean on its GitHub page. Unlike metaFlye, which is a long read metagenome assembler, Canu and Redbean are not designed for metagenomic assembly. However, these assemblers were chosen for comparative analysis as they are frequently used long-read genome assemblers. All of the metaFlye assemblies were polished, using the long reads, with Racon (v1.4.15) [55]. HybridSpades (v3.14.0) [56] and Opera-MS (v0.8.3) [40] (with and without reference genome scaffolding) were used for short read hybrid assembly—short read assembly followed by scaffolding with the long reads. Opera-MS was chosen because it is a metagenome assembler, while HybridSpades was chosen because it is a popular genome assembler. Pilon (v1.23) [57] and ntEdit (v1.3.1) [58] were used for long read hybrid assemblies—long read assembly with metaFlye followed by short read polishing. Each tool was run with 12 cores of 2.70 GHz Intel Xeon E5–2680 processor.

### Assembly statistics

The runtime (user time) of each assembly method on the server was recorded for cumulative batch C_30_ at each enrichment time point. Quast (v5.0.2) [59] was used to report the number of insertion/deletions/mismatches and the NG50 for the C_30_ assembled *L. monocytogenes* contigs with respect to the reference genome.

General quasimetagenomic assembly statistics (total assembly length, the number of contigs, the longest contig, the N50) were collected for every cumulative batch (C_1_ to C_30_ at each enrichment time) using a custom Python script.

### Comparison of the reference genome with the *L. monocytogenes* assembled from the cumulative batches

We estimated the fraction of the reference genome where reads (MiSeq and GridIon) mapped ambiguously, i.e. mapped with the same alignment score to multiple genome locations. The MiSeq reads were mapped with Bowtie2 and the GridIon reads were mapped with MiniMap2. The mean MAPQ score was calculated for each base of the reference genome. Loci with median scores lower than 40 were considered ambiguous [60].

The presence of alleles from the low abundance strain was assessed for the C_30_ assemblies (across enrichment times) with Snippy by aligning the assemblies to the reference genome and cross-referencing the variant loci identified when looking for multiple strains.

The *L. monocytogenes* contigs assembled from each cumulative batch (C_1_ to C_30_ at each enrichment time) were assessed for accuracy with respect to the reference genome. Accuracy was assessed by measuring the BLAST distance (a measure of sequence similarity) between the predicted genes (both the core and complete set of genes) of the reference and the *L. monocytogenes* metagenome-assembled genomes. We define the BLAST distance as the number of mismatches, insertions, and deletions in the BLAST alignment between the reference genes and the assembled genes. Preferably, the edit distance between the reference genes and the genes found in the assemblies would have been calculated, but correctly identifying the entire length of genes, especially in noisy long read assemblies, is difficult; instead, the BLAST distance forms an approximation of the edit distance.

If the *L. monocytogenes* genome assembled from a sample comprised a single contig, the synteny of the core genes was compared to that in the reference.

### Taxonomic classification

The contigs from the MegaHit (short read) assemblies and metaFlye (long read) assemblies (from each cumulative batch at every enrichment time) were taxonomically classified with Kraken (v1.1.1) [61] and the MiniKraken database using default settings. A species was considered present if ≥5000 nt of contigs were annotated as that species.

The proportion of reads mapped to the *L. monocytogenes* genome was used as the relative abundance of *L. monocytogenes* in the samples.

## Supplementary Information

**Additional file 1: Supplementary Figure 1.** Read length distributions for long reads that mapped to the *Listeria monocytogenes* reference genome versus those that did not.

**Additional file 2: Supplementary Figure 2.** GC content distributions for long reads that mapped to the *Listeria monocytogenes* reference genome and those that did not.

**Additional file 3: Supplementary Figure 3.** Runtimes for the assembly approaches in minutes when assembling cumulative batch 30 from each of the enrichment time points. (Abbreviations: SR = short read, LR = long read, HY = hybrid).

**Additional file 4: Supplementary Figure 4.** Total assembly length versus the total number of base pairs sequenced per cumulative batch at each of the enrichment time points for each assembly approach. Sometimes the results for Canu, Redbean and metaFlye overlap as do Opera-MS (NoRef) and Opera-Ms (RefCluster). (Abbreviations: SR = short read, LR = long read, HY = hybrid).

**Additional file 5: Supplementary Figure 5.** Number of contigs versus the total number of base pairs sequenced per cumulative batch at each of the enrichment time points for each assembly approach. metaFlye+Racon, metaFlye+Racon+Pilon, and metaFlye+Racon+ntEdit are obscured by the line for metaFlye in each of the plots. (Abbreviations: SR = short read, LR = long read, HY = hybrid).

**Additional file 6: Supplementary Figure 6.** N50 versus the total number of base pairs sequenced per cumulative batch at each of the enrichment time points for each assembly approach. metaFlye+Racon, metaFlye+Racon+Pilon, and metaFlye+Racon+ntEdit are obscured by the line for metaFlye in each of the plots. (Abbreviations: SR = short read, LR = long read, HY = hybrid).

**Additional file 7: Supplementary Figure 7.** The longest contig assembled versus the total number of base pairs sequenced per cumulative batch at each of the enrichment time points for each assembly approach. metaFlye+Racon, metaFlye+Racon+Pilon, and metaFlye+Racon+ntEdit are obscured by the line for metaFlye in each of the plots. (Abbreviations: SR = short read, LR = long read, HY = hybrid).

## Data Availability

The short and long reads are available on NCBI in Bioproject PRJNA630588. The *L. monocytogenes* reference genome is available on Genbank under accession NZ_CP016213.1. The code and data used for our analysis is available on GitHub at https://github.com/SethCommichaux/Long_read_short_read_comparison.
