# Supplementary figures and images for: Evaluating the accuracy of Listeria monocytogenes assemblies from quasimetagenomic samples using long and short reads
Source: BMC Genomics. 2021 May 26;22:389. doi: 10.1186/s12864-021-07702-2 (PMC8157722; doi:10.1186/s12864-021-07702-2)

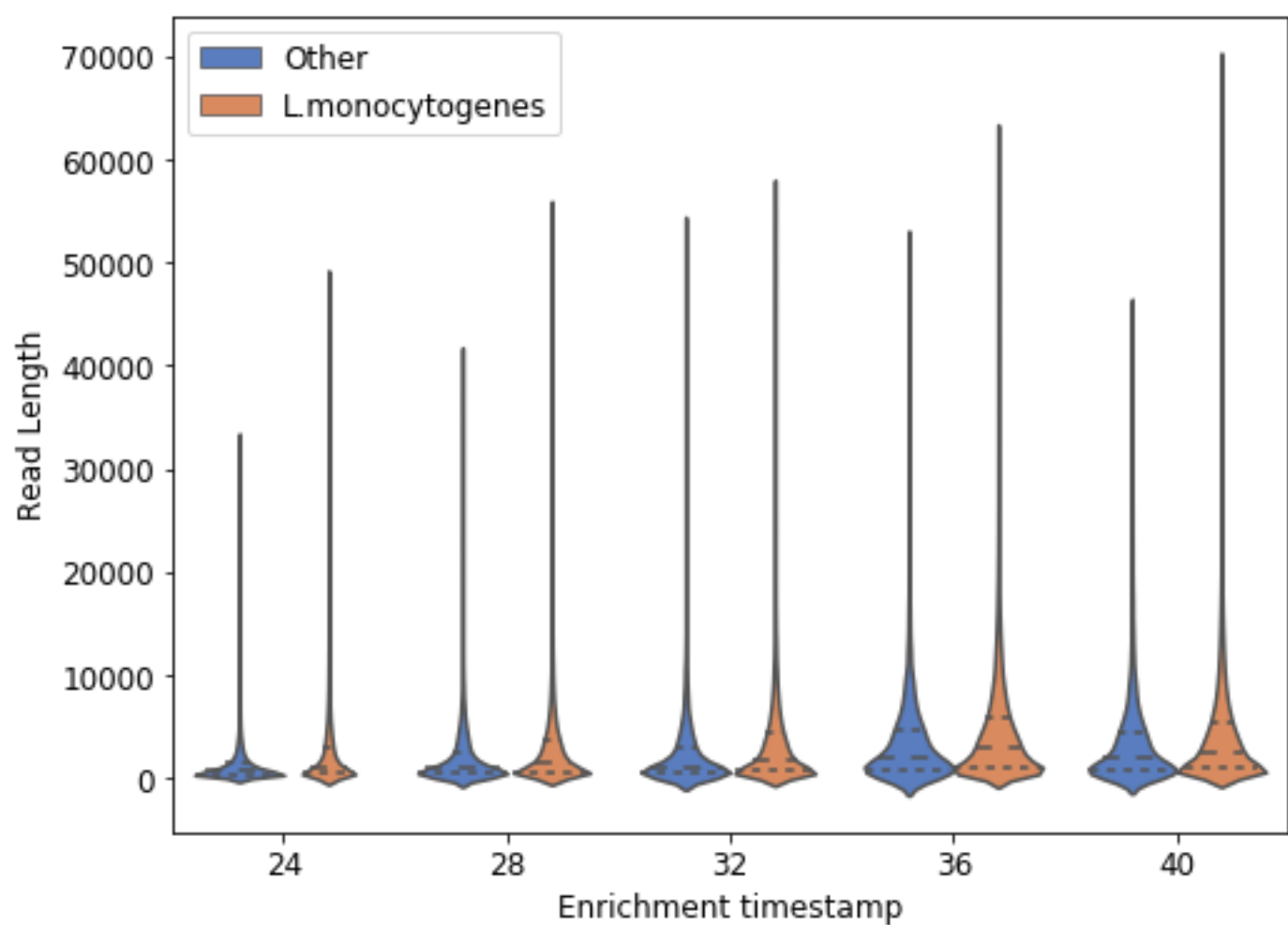

Supplement: Supplementary file 1 — Additional file 1: Supplementary Figure 1. Read length distributions for long reads that mapped to the Listeria monocytogenes reference genome versus those that did not. [file 12864_2021_7702_MOESM1_ESM.pdf]

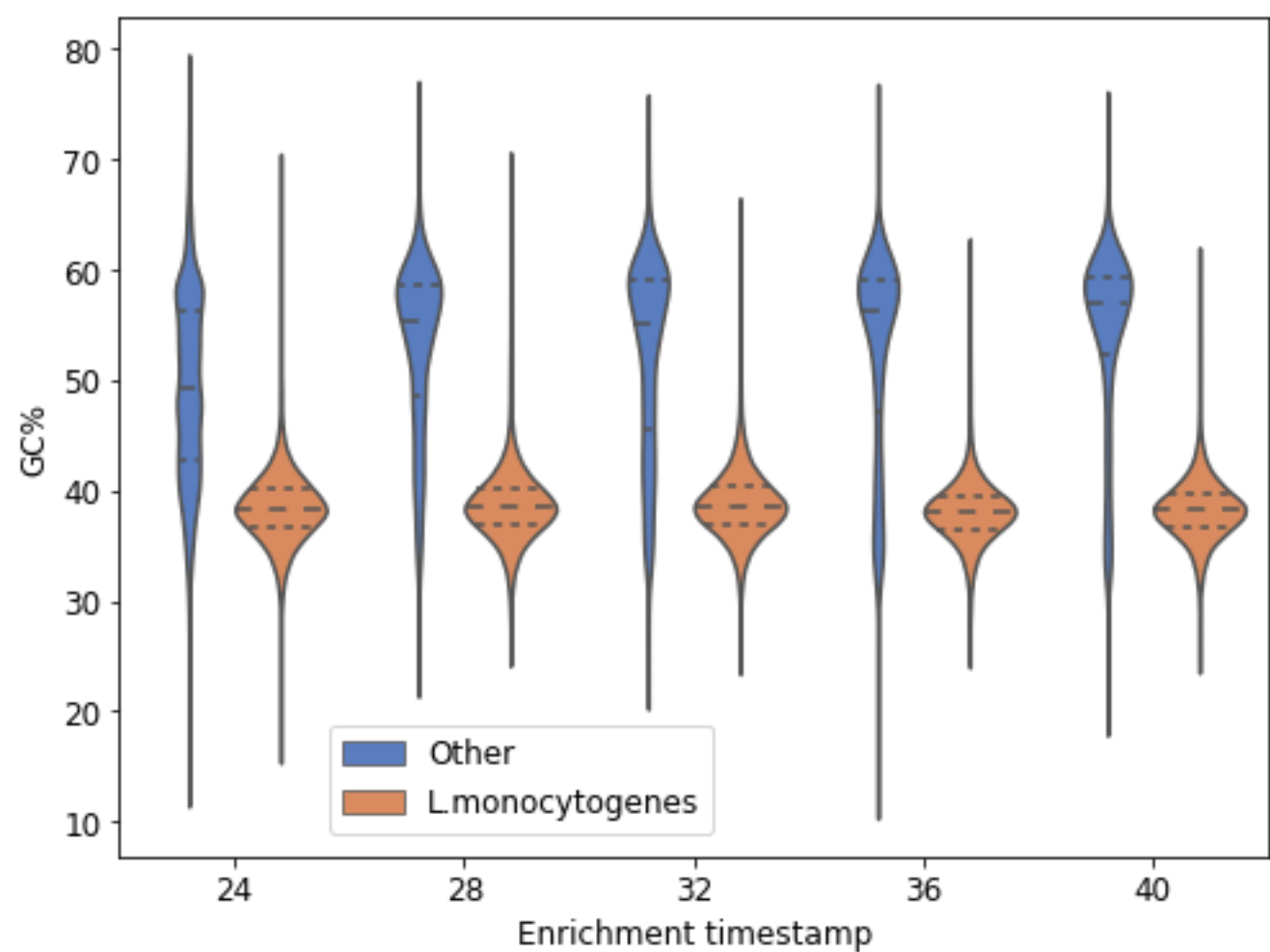

Supplement: Supplementary file 2 — Additional file 2: Supplementary Figure 2. GC content distributions for long reads that mapped to the Listeria monocytogenes reference genome and those that did not. [file 12864_2021_7702_MOESM2_ESM.pdf]

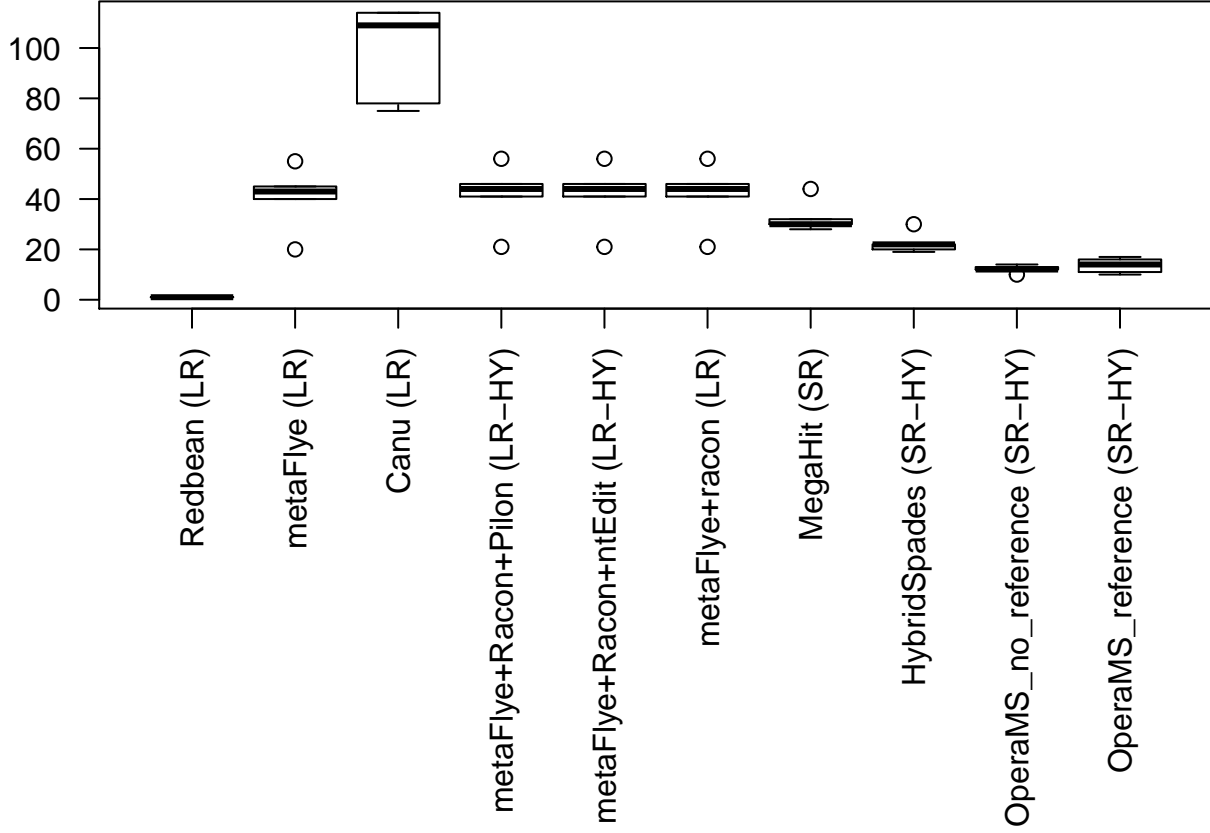

Supplement: Supplementary file 3 — Additional file 3: Supplementary Figure 3. Runtimes for the assembly approaches in minutes when assembling cumulative batch 30 from each of the enrichment time points. (Abbreviations: SR = short read, LR = long read, HY = hybrid). [file 12864_2021_7702_MOESM3_ESM.pdf]

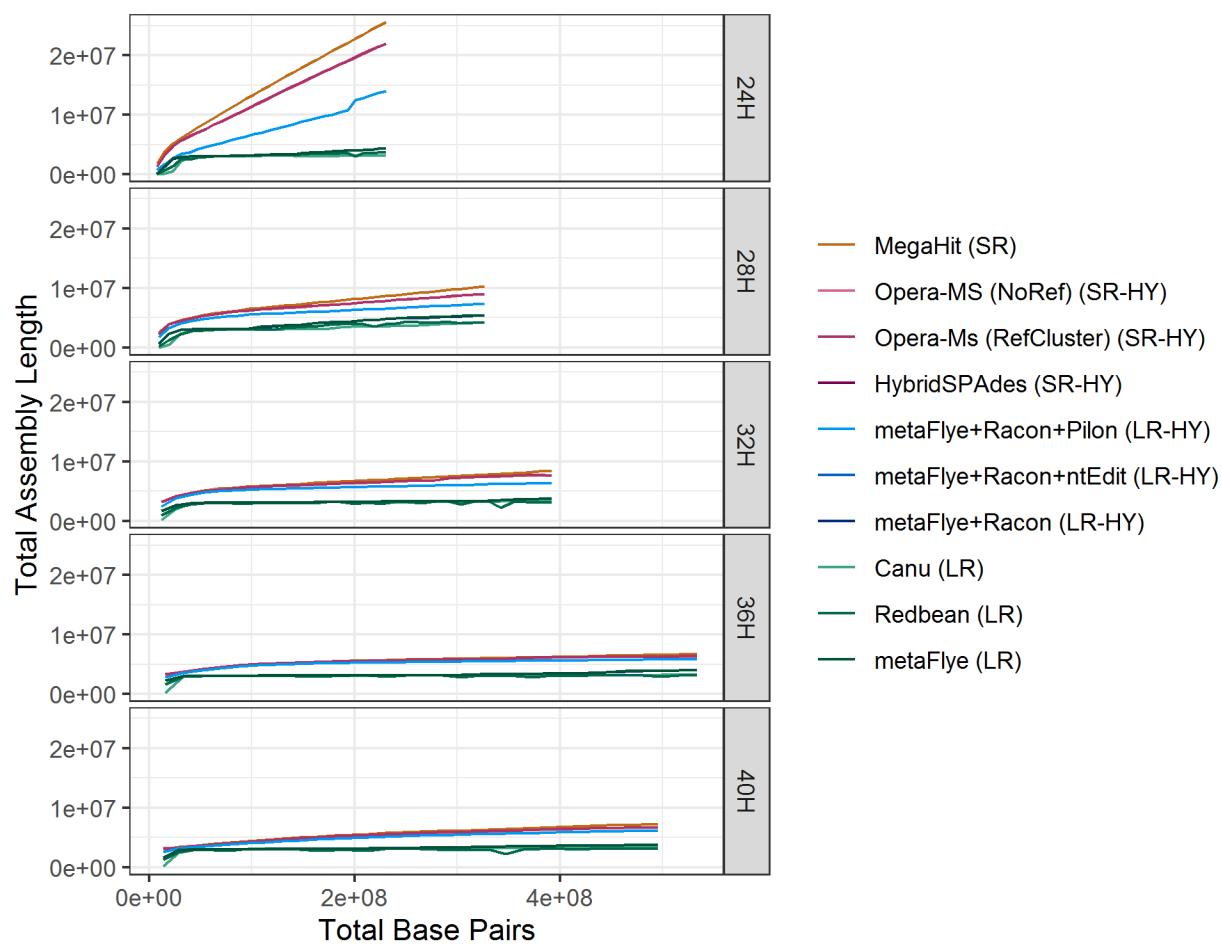

Supplement: Supplementary file 4 — Additional file 4: Supplementary Figure 4. Total assembly length versus the total number of base pairs sequenced per cumulative batch at each of the enrichment time points for each assembly approach. Sometimes the results for Canu, Redbean and metaFlye overlap as do Opera-MS (NoRef) and Opera-Ms (RefCluster). (Abbreviations: SR = short read, LR = long read, HY = hybrid). [file 12864_2021_7702_MOESM4_ESM.pdf]

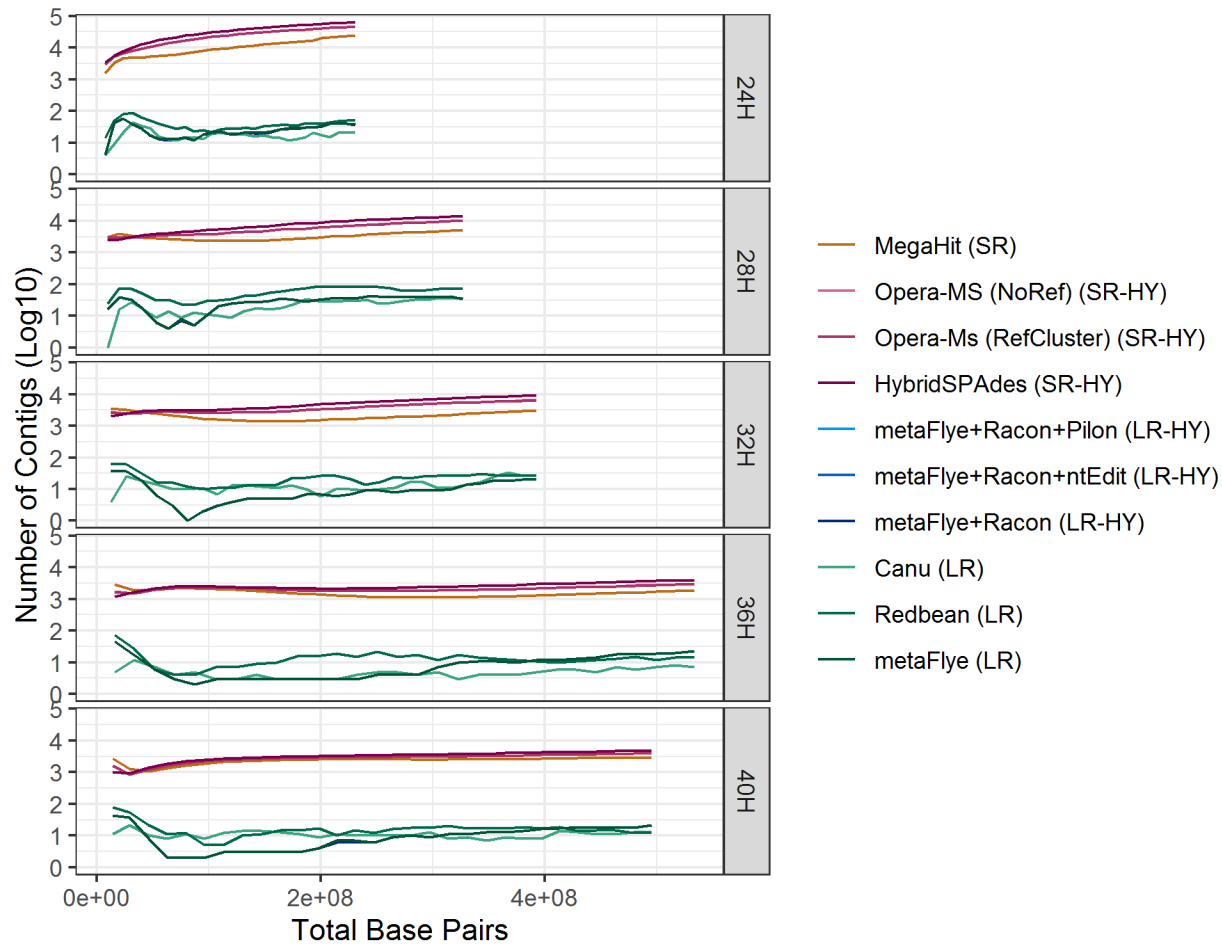

Supplement: Supplementary file 5 — Additional file 5: Supplementary Figure 5. Number of contigs versus the total number of base pairs sequenced per cumulative batch at each of the enrichment time points for each assembly approach. metaFlye+Racon, metaFlye+Racon+Pilon, and metaFlye+Racon+ntEdit are obscured by the line for metaFlye in each of the plots. (Abbreviations: SR = short read, LR = long read, HY = hybrid). [file 12864_2021_7702_MOESM5_ESM.pdf]

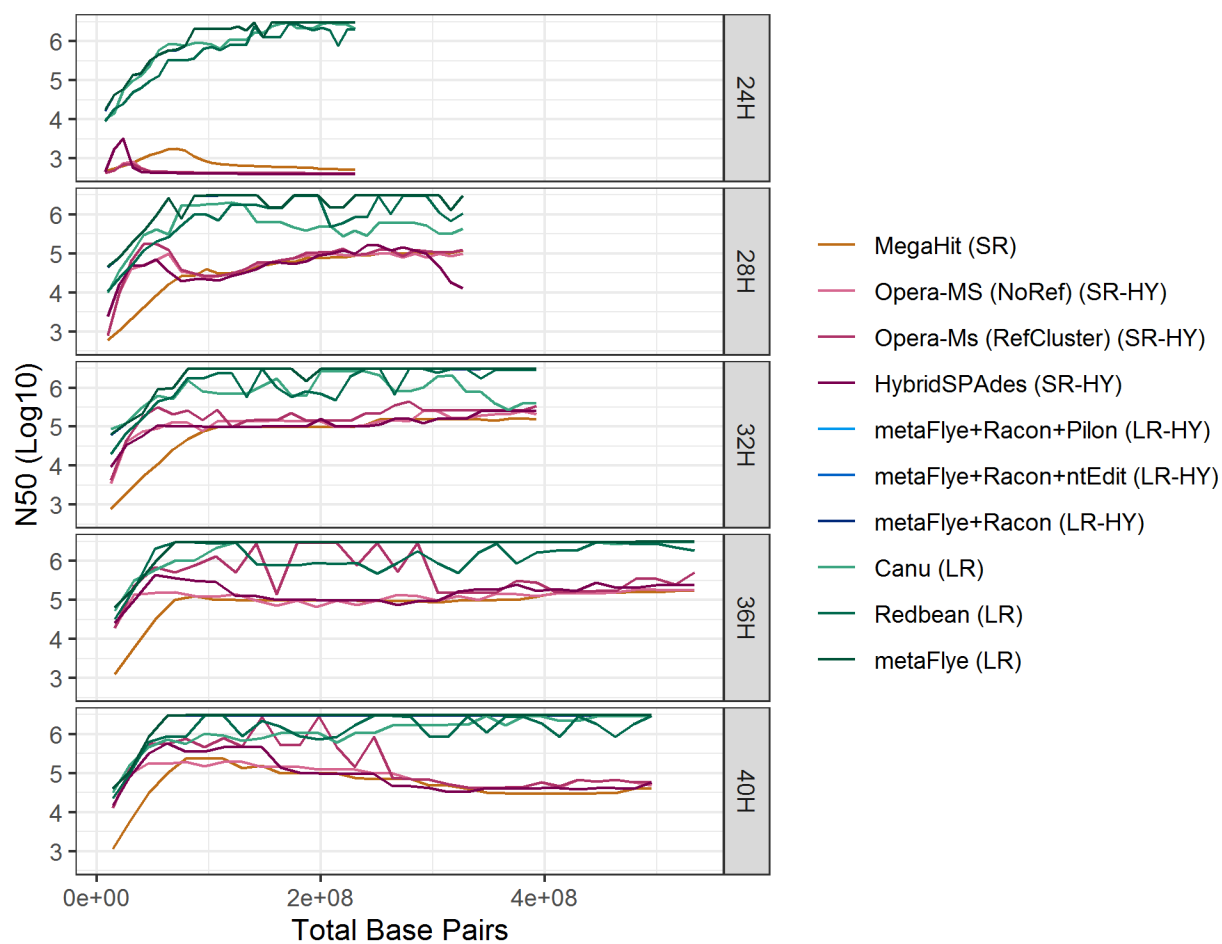

Supplement: Supplementary file 6 — Additional file 6: Supplementary Figure 6. N50 versus the total number of base pairs sequenced per cumulative batch at each of the enrichment time points for each assembly approach. metaFlye+Racon, metaFlye+Racon+Pilon, and metaFlye+Racon+ntEdit are obscured by the line for metaFlye in each of the plots. (Abbreviations: SR = short read, LR = long read, HY = hybrid). [file 12864_2021_7702_MOESM6_ESM.pdf]

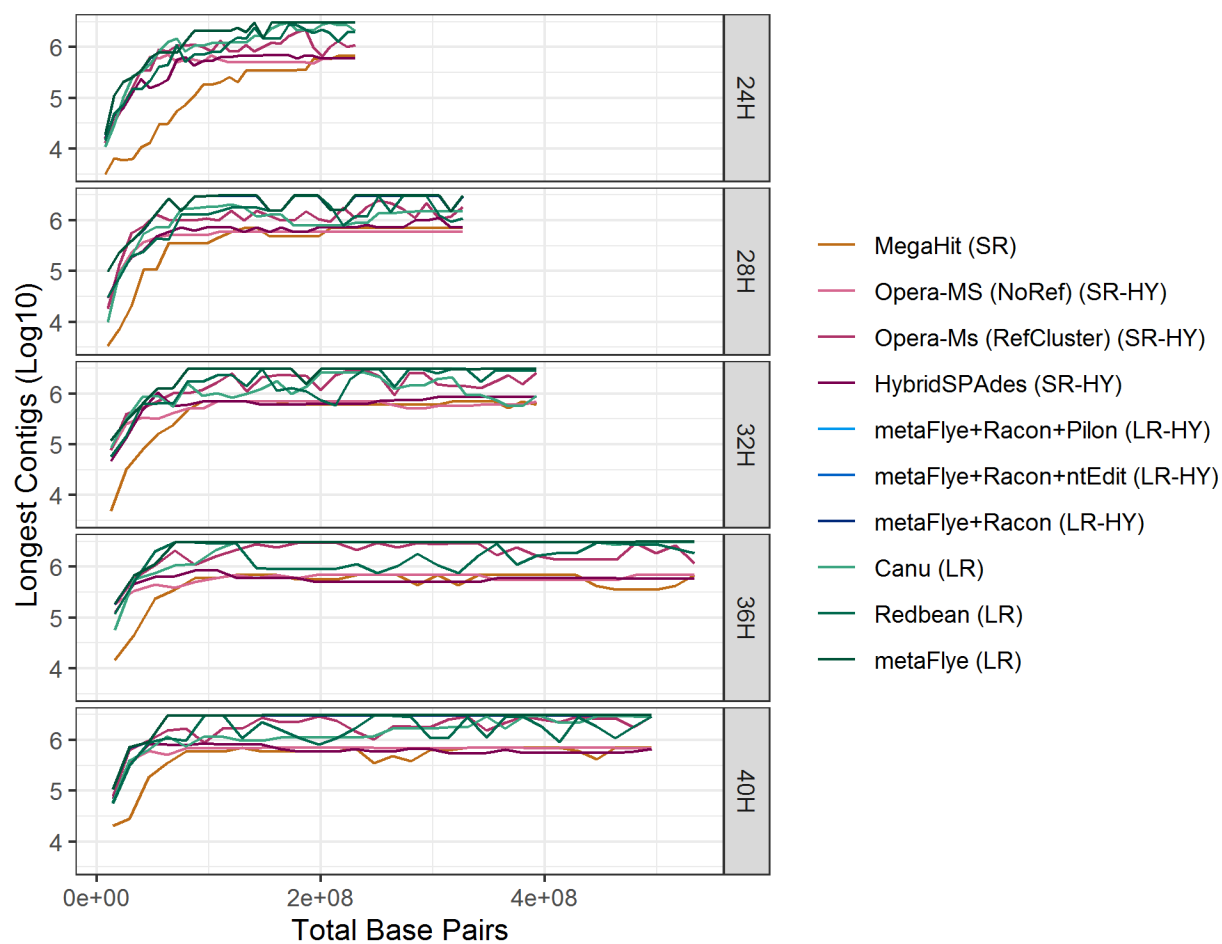

Supplement: Supplementary file 7 — Additional file 7: Supplementary Figure 7. The longest contig assembled versus the total number of base pairs sequenced per cumulative batch at each of the enrichment time points for each assembly approach. metaFlye+Racon, metaFlye+Racon+Pilon, and metaFlye+Racon+ntEdit are obscured by the line for metaFlye in each of the plots. (Abbreviations: SR = short read, LR = long read, HY = hybrid). [file 12864_2021_7702_MOESM7_ESM.pdf]
